# Impact of COVID-19 Nonpharmaceutical Interventions on Pneumococcal Carriage Prevalence and Density in Vietnam

**DOI:** 10.1128/spectrum.03615-22

**Published:** 2023-01-16

**Authors:** Monica Larissa Nation, Sam Manna, Hau Phuc Tran, Cattram Duong Nguyen, Le Thi Tuong Vy, Doan Y. Uyen, Tran Linh Phuong, Vo Thi Trang Dai, Belinda Daniela Ortika, Ashleigh Christina Wee-Hee, Jemima Beissbarth, Jason Hinds, Kathryn Bright, Heidi Smith-Vaughan, Thuong Vu Nguyen, Kim Mulholland, Beth Temple, Catherine Satzke

**Affiliations:** a Infection and Immunity, Murdoch Children's Research Institute, Parkville, Victoria, Australia; b Department of Paediatrics, The University of Melbourne, Parkville, Victoria, Australia; c Department of Microbiology and Immunology at the Peter Doherty Institute for Infection and Immunity, The University of Melbourne, Parkville, Victoria, Australia; d Department of Disease Control and Prevention, Pasteur Institute of Ho Chi Minh City, Ho Chi Minh City, Vietnam; e Clinical Research Center, Pasteur Institute of Ho Chi Minh City, Ho Chi Minh City, Vietnam; f Department of Microbiology and Immunology, Pasteur Institute of Ho Chi Minh City, Ho Chi Minh City, Vietnam; g Child Health Division, Menzies School of Health Research, Charles Darwin University, Darwin, Northern Territory, Australia; h Institute for Infection and Immunity, St. George's University of London, London, England, United Kingdom; i BUGS Bioscience, London Bioscience Innovation Centre, London, England, United Kingdom; j Epidemiology and Population Health, London School of Hygiene and Tropical Medicine, London, England, United Kingdom; k Global and Tropical Health Division, Menzies School of Health Research, Charles Darwin University, Casuarina, Northern Territory, Australia; Griffith University

**Keywords:** *Streptococcus pneumoniae*, density, carriage, pneumococcal, Vietnam, COVID-19, nonpharmaceutical intervention

## Abstract

Nonpharmaceutical interventions (NPIs) implemented to contain SARS-CoV-2 have decreased invasive pneumococcal disease. Previous studies have proposed the decline is due to reduced pneumococcal transmission or suppression of respiratory viruses, but the mechanism remains unclear. We undertook a secondary analysis of data collected from a clinical trial to evaluate the impact of NPIs on pneumococcal carriage and density, drivers of transmission and disease, during the COVID-19 pandemic in Ho Chi Minh City, Vietnam. Nasopharyngeal samples from children aged 24 months were assessed in three periods — one pre-COVID-19 period (*n* = 1,537) and two periods where NPIs were implemented with increasing stringency (NPI period 1 [NPI-1, *n* = 307], and NPI period 2 [NPI-2, *n* = 262]). Pneumococci were quantified using *lytA* quantitative PCR and serotyped by DNA microarray. Overall, capsular, and nonencapsulated pneumococcal carriage and density were assessed in each NPI period compared with the pre-COVID-19 period using unadjusted log-binomial and linear regression. Pneumococcal carriage was generally stable after the implementation of NPIs. In contrast, overall pneumococcal carriage density decreased by 0.44 log_10_ genome equivalents/mL (95% confidence interval [CI]: 0.19 to 0.69) in NPI-1 and by 0.84 log_10_ genome equivalents/mL (95% CI: 0.55 to 1.13) in NPI-2 compared with the pre-COVID-19 period. Reductions in overall pneumococcal density were driven by reductions in capsular pneumococci, with no corresponding reduction in nonencapsulated density. As higher pneumococcal density is a risk factor for disease, the decline in density provides a plausible explanation for the reductions in invasive pneumococcal disease that have been observed in many countries in the absence of a substantive reduction in pneumococcal carriage.

**IMPORTANCE** The pneumococcus is a major cause of mortality globally. Implementation of NPIs during the COVID-19 pandemic led to reductions in invasive pneumococcal disease in many countries. However, no studies have conducted a fully quantitative assessment on the impact of NPIs on pneumococcal carriage density, which could explain this reduction. We evaluated the impact of COVID-19 NPIs on pneumococcal carriage prevalence and density in 2,106 children aged 24 months in Vietnam and found pneumococcal carriage density decreased up to 91.5% after NPI introduction compared with the pre-COVID-19 period, which was mainly attributed to capsular pneumococci. Only a minor effect on carriage prevalence was observed. As respiratory viruses are known to increase pneumococcal carriage density, transmission, and disease, this work suggests that interventions targeting respiratory viruses may have the added benefit of reducing invasive pneumococcal disease and explain the reductions observed following NPI implementation.

## INTRODUCTION

Streptococcus pneumoniae (the pneumococcus) is a major cause of disease, including pneumonia, meningitis, and sepsis. The bacterium commonly colonizes the nasopharynx and carriage is a prerequisite for pneumococcal transmission and disease (1). During the COVID-19 pandemic, the implementation of nonpharmaceutical interventions (NPIs) such as mask wearing, stay-at-home orders, and physical distancing have been critical for the containment of SARS-CoV-2. Introduction of NPIs have been associated with declines in other respiratory pathogens and infectious diseases, including influenza, respiratory syncytial virus (RSV), pneumonia, and invasive pneumococcal disease (IPD) (2–4). It has been proposed that the reduction in IPD may be due to reduced pneumococcal transmission (3), but there are limited data to support this. Four studies have examined the impact of NPIs on pneumococcal carriage prevalence, with varying results (4–7). A study in Belgium found no difference in carriage prevalence pre- and postimplementation of NPIs in children attending daycare. A study in an outpatient health care facility in Serbia found an increase in pneumococcal carriage in children during the COVID-19 pandemic compared with the carriage prevalence in the early pandemic period (7). In contrast, a study in Israel found a reduction in pneumococcal carriage prevalence in one of the two NPI periods assessed, along with a decline in the prevalence of respiratory viruses that was temporally associated with a decline in IPD (4). A study in France found that pneumococcal carriage rates remained stable but a reduction in influenza-like illness and RSV was associated with a 63% decline in IPD (6). Furthermore, the study in France estimated that 93% of the decrease in IPD incidence was associated with decreases in the number of influenza and RSV cases. Collectively, these studies suggest pneumococcal carriage has generally remained stable during the COVID-19 pandemic, while there has been a decline in some circulating respiratory viruses.

Some respiratory viruses increase pneumococcal carriage density, which contributes to transmission and disease (8–13). Therefore, we hypothesized that reductions in IPD associated with NPIs may be due to reductions in pneumococcal carriage density rather than prevalence. To date, no studies have conducted a fully quantitative assessment of the impact of NPIs on pneumococcal carriage density, which may provide the missing link between temporal reductions in circulating respiratory viruses and IPD. Here, we aim to assess the impact of NPIs during the COVID-19 pandemic on pneumococcal carriage prevalence and density in children in Vietnam.

## RESULTS

**FIG 1:**
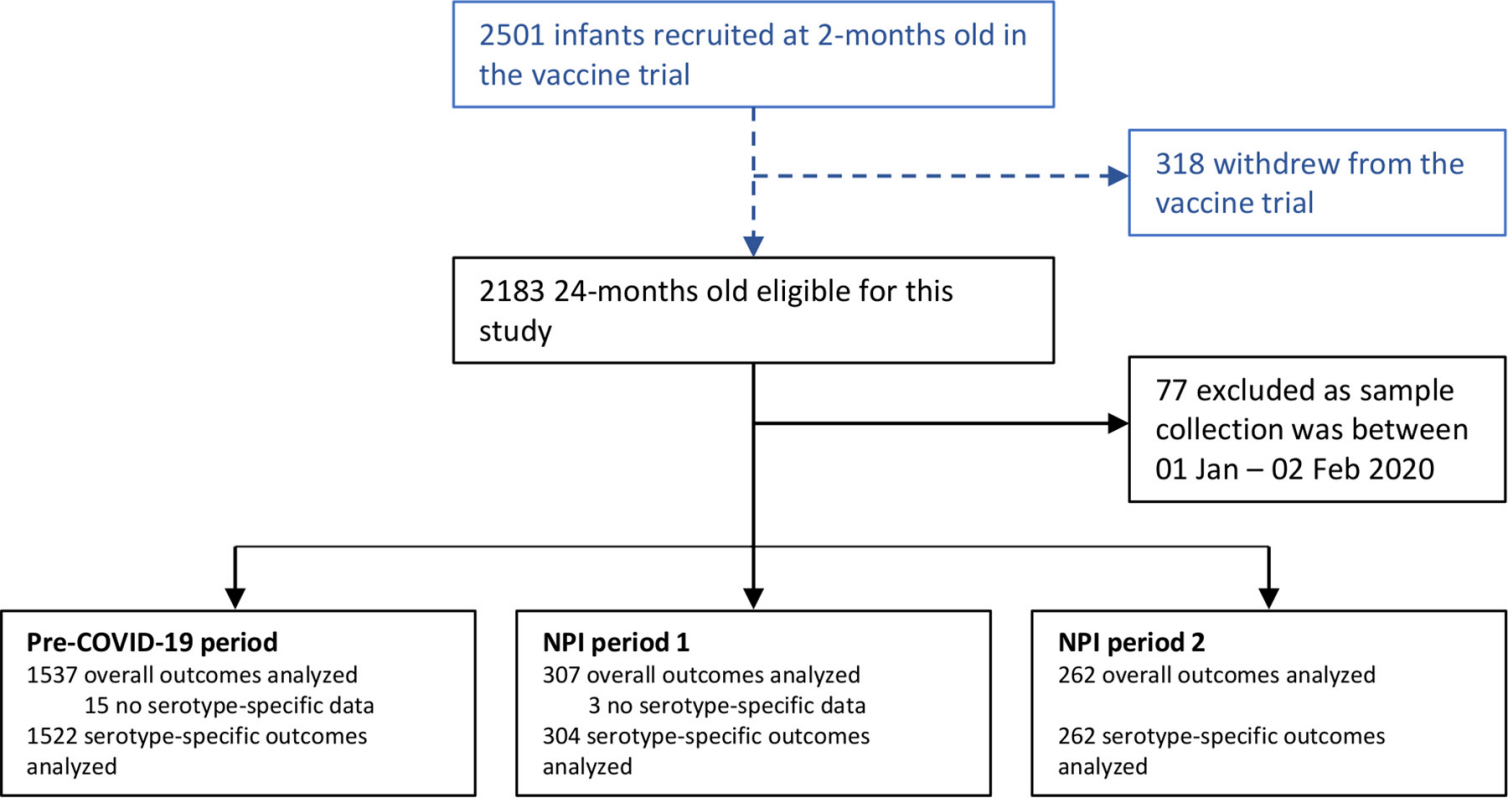
Participant flow diagram. Participants were excluded from all analyses if samples were collected between 1 January to 2 February 2020, as preventative measures may have been implemented before the nonpharmaceutical interventions (NPI) were officially introduced (*n* = 77) or were excluded from serotype-specific analyses if serotype-specific data were not available (*n* = 18).

Of the 2,501 participants enrolled in the trial, 318 withdrew and do not contribute data to this analysis (Fig. 1). Between 25 December 2018 and 18 June 2020, 2,183 nasopharyngeal samples were collected from children aged approximately 24 months old. Over the three periods, 2,106 nasopharyngeal samples were included in this analysis (*n* = 1,537 in pre-COVID-19, *n* = 307 in NPI-1, and *n* = 262 in NPI-2). Seventy-seven samples were collected between 1 January and 2 February 2020 and were excluded from analysis as preventative measures may have been implemented before such policies were officially introduced. Eighteen samples were excluded from serotype-specific analyses as no serotyping data were available.

Participant characteristics were similar across the three periods for sex, current breastfeeding, household size, the number of children under 5 years of age in the household (including the participant), number of pneumococcal conjugate vaccine (PCV) doses received, and the type of PCV received (Table 1). Children were older, reported fewer respiratory symptoms, and used fewer antibiotics in both NPI periods compared with the pre-COVID-19 period. Samples were collected across both seasons in the pre-COVID-19 period, while all samples in NPI period 1 were collected in the dry season and all samples in NPI period 2 were collected in the rainy season. There were differences in the proportion of participants from each district, as recruitment commenced at different times in the districts.

**TABLE 1:** Participant characteristics at the 24-month visit^*a*^

Characteristics	Pre-COVID-19 *n* = 1,537	NPI period 1 *n* = 307	NPI period 2 *n* = 262	*P* value^*b*^
Age in months (median [range])	24.1 (24.0–28.5)	24.1 (24.0–28.7)	25.0 (24.0–30.8)	<0.001
Sex, female	740 (48.1%)	148 (48.2%)	131 (50.0%)	0.860
District				<0.001
4	663 (43.1%)	88 (28.7%)	74 (28.2%)	
7	381 (24.8%)	104 (33.9%)	68 (26.0%)	
8	493 (32.1%)	115 (37.5%)	120 (45.8%)	
Household size (median [range])^*c*^	6 (3–34)	6 (3–26)	6 (3–18)	1.000
No. of children under 5 in household^*c*^^,^^*d*^				0.671
1	897 (58.5%)	193 (62.9%)	153 (58.4%)	
2	497 (32.4%)	91 (29.6%)	84 (32.1%)	
≥3	140 (9.1%)	23 (7.5%)	25 (9.5%)	
Season^*e*^				<0.001
Dry	513 (33.4%)	307 (100%)	0 (0%)	
Rainy	1,024 (66.6%)	0 (0%)	262 (100%)	
Current breastfeeding	120 (7.8%)	19 (6.2%)	14 (5.3%)	0.268
Current URTI symptoms	250 (16.3%)	14 (4.6%)	7 (2.7%)	<0.001
Antibiotic use in past 2 wks	175 (11.4%)	8 (2.6%)	2 (0.8%)	<0.001
Current antibiotic use	62 (4.0%)	5 (1.6%)	0 (0%)	0.001
Doses of PCV received^*f*^				0.820
0	540 (35.1%)	110 (35.8%)	87 (33.2%)	
1	498 (32.4%)	91 (29.6%)	89 (34.0%)	
2	499 (32.5%)	106 (34.5%)	86 (32.8%)	
If PCV received, PCV10	486 (48.7%)	101 (51.3%)	88 (50.3%)	0.780

a Data are *n* (%) unless specified. URTI, upper respiratory tract infection; PCV, pneumococcal conjugate vaccine; PCV10, 10-valent PCV.

b *P* values are comparisons across all time periods based on chi-squared test (for comparisons of proportions), or quantile regression with bootstrapped standard errors (for comparisons of medians).

c Missing data for *n* = 3 participants in the pre-COVID-19 period.

d Including the participant.

e Ho Chi Minh City has a Tropical Savannah climate (27) consisting of a dry season (November to April) and a rainy season (May to October).

f Participants received either PCV10 or PCV13.

Of the 2,106 children assessed, 481 (22.8%) carried pneumococci. Multiple serotype carriage was infrequent (39/2088; 1.9%) and was similar across the three periods (25/1522 (1.6%) in the pre-COVID-19 period, 8/304 (2.6%) in NPI-1, and 6/262 (2.3%) in NPI-2).

Overall pneumococcal carriage prevalence was 23.6% (363/1,537), 22.8% (70/307), and 18.3% (48/262) in the pre-COVID-19, NPI-1, and NPI-2 periods, respectively (Table 2). There was no difference in overall pneumococcal carriage prevalence in NPI-1 compared with the pre-COVID-19 period (prevalence ratio [PR]: 0.97; 95% confidence interval [CI]: 0.77 to 1.21) (Table 2 and Fig. S2 in the supplemental material). There was some evidence of a reduction in overall carriage prevalence in NPI-2 compared with the pre-COVID-19 period (PR: 0.78; 95% CI: 0.59 to 1.02; *P* value = 0.066). This was driven by a lower point estimate for nonencapsulated carriage prevalence, with wide confidence intervals and a small number of observations. Neither NPI period was associated with changes in capsular carriage prevalence compared with the pre-COVID-19 period.

**TABLE 2:** Pneumococcal carriage prevalence and prevalence ratios in periods with nonpharmaceutical interventions (NPIs) compared with the pre-COVID-19 period^*a*^

Carriage	Prevalence			NPI-1 vs pre-COVID-19		NPI-2 vs pre-COVID-19	
	Pre-COVID-19	NPI-1	NPI-2	Prevalence ratio (95% CI)	*P* value	Prevalence ratio (95% CI)	*P* value
Overall	363/1,537 (23.6%)	70/307 (22.8%)	48/262 (18.3%)	0.97 (0.77 to 1.21)	0.759	0.78 (0.59 to 1.02)	0.066
Capsular^*b*^	269/1,522 (17.7%)	52/304 (17.1%)	40/262 (15.3%)	0.97 (0.74 to 1.27)	0.812	0.86 (0.64 to 1.17)	0.347
Nonencapsulated^*b*^	95/1,522 (6.2%)	20/304 (6.6%)	10/262 (3.8%)	1.05 (0.66 to 1.68)	0.825	0.61 (0.32 to 1.16)	0.131

a COVID-19, coronavirus disease 2019; NPI-1, nonpharmaceutical intervention period 1; NPI-2, nonpharmaceutical intervention period 2; 95% CI, 95% confidence interval.

b Serotype-specific data were not available for *n* = 18 samples (*n* = 15 pre-COVID-19, *n* = 3 NPI-1).

**FIG 2:**
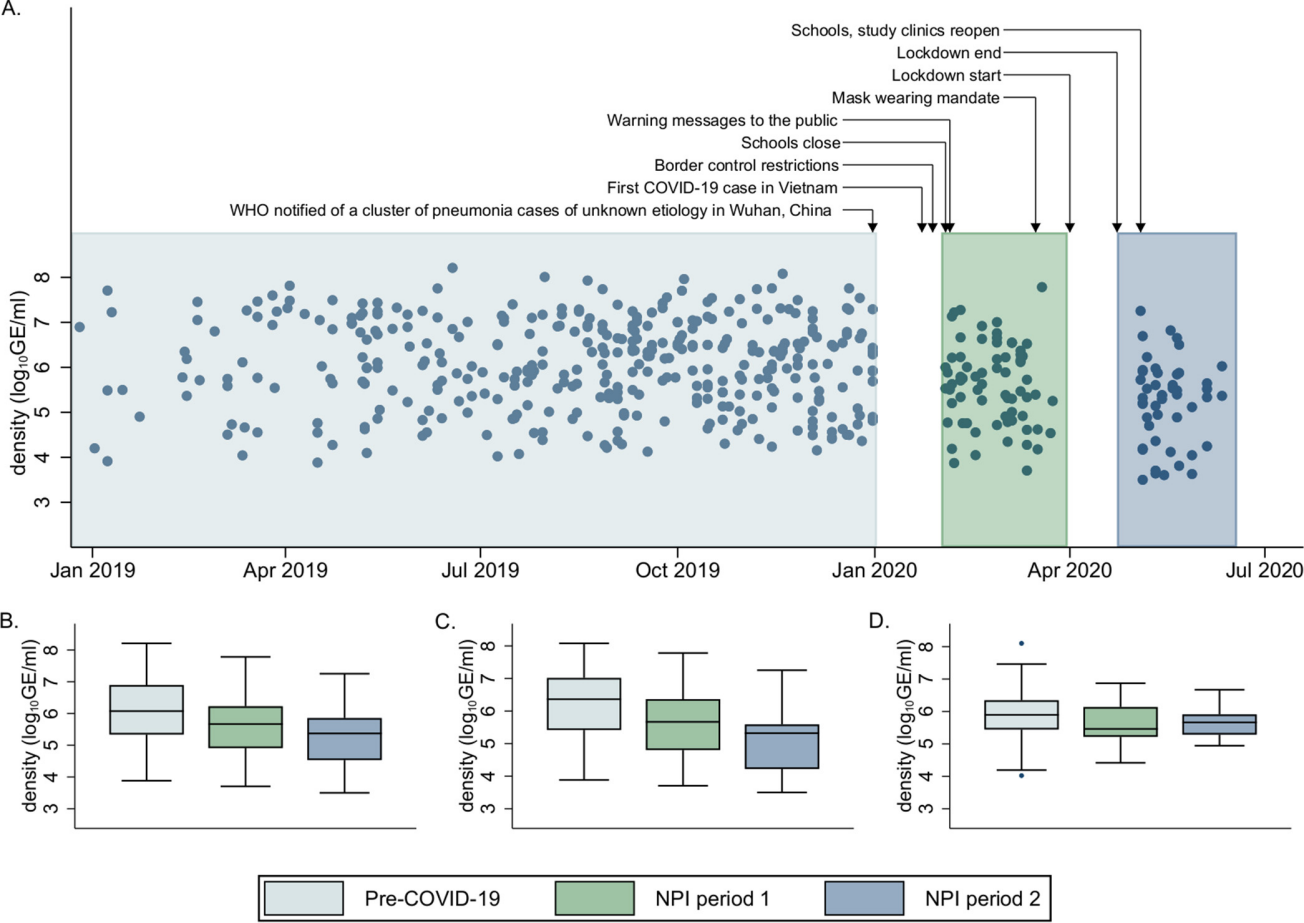
Nasopharyngeal carriage density (log_10_ genome equivalents per mL) among pneumococcal carriers. (A) Overall pneumococcal density from 2019 to 2020 with key events and nonpharmaceutical interventions (NPIs) indicated. Box plots of (B) overall, (C) capsular, and (D) nonencapsulated pneumococcal density. Boxes depict interquartile range (IQR) with a central line for the median. Data points further than the 25^th^/75^th^ percentile plus 1.5 times the IQR are plotted as individual points. COVID-19 = coronavirus disease 2019.

Overall and capsular pneumococcal carriage density decreased from pre-COVID-19 to NPI-1, with a further reduction in NPI-2 (Fig. 2 and Table 3). Overall pneumococcal carriage density decreased by 63.7% (reduction of 0.44 log_10_ genome equivalents per mL [GE/mL]; 95% CI: 0.19 to 0.69) in NPI-1 and by 85.5% (reduction of 0.84 log_10_ GE/mL; 95% CI: 0.55 to 1.13) in NPI-2 compared with the pre-COVID-19 period (Table 3 and Fig. S2). Reductions in overall pneumococcal carriage density were driven by decreases in capsular pneumococcal carriage density, which decreased by 70.5% from pre-COVID-19 to NPI-1 (reduction of 0.53 log_10_ GE/mL; 95% CI: 0.23 to 0.83), and by 91.5% from pre-COVID-19 to NPI-2 (reduction of 1.07 log_10_ GE/mL; 95% CI: 0.74 to 1.41). There was no corresponding reduction in nonencapsulated pneumococcal carriage density in NPI-1 (percentage change = −48.7%; difference in means = −0.29 log_10_ GE/mL; 95% CI: 0.64 to 0.05) or NPI-2 (percentage change = −38.3%; difference in means = −0.21 log_10_ GE/mL; 95% CI: −0.68 to 0.26) compared with the pre-COVID-19 period.

**TABLE 3:** Pneumococcal carriage density means and difference in means in periods with nonpharmaceutical interventions (NPIs) compared with the pre-COVID-19 period^*a*^

Density^*b*^	*n*	Mean (SD)			NPI-1 vs pre-COVID-19		NPI-2 vs pre-COVID-19	
		Pre-COVID-19	NPI-1	NPI-2	Difference in means (95% CI)	*P* value	Difference in means (95% CI)	*P* value
Overall	481	6.07 (0.99)	5.63 (0.90)	5.23 (0.92)	−0.44 (−0.69 to −0.19)	0.001	−0.84 (−1.13 to −0.55)	<0.001
Capsular^*c*^	361	6.18 (1.01)	5.65 (0.95)	5.11 (0.95)	−0.53 (−0.83 to −0.23)	0.001	−1.07 (−1.41 to −0.74)	<0.001
Nonencapsulated^*c*^	125	5.90 (0.74)	5.60 (0.63)	5.69 (0.52)	−0.29 (−0.64 to 0.05)	0.098	−0.21 (−0.68 to 0.26)	0.381

a NPI-1, nonpharmaceutical intervention period 1; NPI-2, nonpharmaceutical intervention period 2; COVID-19, coronavirus disease 2019; SD, standard deviation; 95% CI, 95% confidence interval.

b Assessed in pneumococcal carriers only and reported in log_10_ genome equivalents per mL.

c Serotype-specific data were not available for *n* = 18 samples (pre-COVID-19, *n* = 15; NPI-1, *n* = 3).

Overall, we found no clear patterns of serotype-specific changes in pneumococcal carriage prevalence by NPI period (Fig. S3A). Pneumococcal carriage density was generally lower in NPI-2 for most serotypes but as numbers were small, we did not conduct formal analyses comparing individual serotypes (Fig. S3B).

Similar patterns were observed in the direct analyses adjusting for the intermediate variables district of residence and season (Table S1), when analyses were restricted to participants without URTI symptoms (Table S2), and when analyses were restricted to unvaccinated participants (Table S3). There was little evidence of a reduction in pneumococcal carriage prevalence in NPI-2 compared with the pre-COVID-19 period in any of the additional analyses.

## DISCUSSION

Following the implementation of NPIs for the containment of SARS-CoV-2, a decline in disease caused by other respiratory pathogens, including pneumococci, has been observed in multiple countries. The decline in IPD has been attributed to reductions in pneumococcal transmission (3); however, four studies (including this one) have found pneumococcal carriage prevalence was generally stable after the implementation of NPIs (4–6). We found some evidence of a 22% reduction in overall pneumococcal carriage prevalence in one of the periods after NPI implementation, which was driven by a decrease in the prevalence of nonencapsulated pneumococci. As nonencapsulated pneumococci are infrequently implicated in IPD (14), it is unlikely that a change in nonencapsulated carriage prevalence would translate to a substantial decline in IPD as observed in many countries. Studies in Belgium and France found no substantive reductions in pneumococcal carriage pre- and post-NPIs, although similarly to our study, the study in Israel found pneumococcal carriage prevalence was lower in one of the two time periods assessed (4–6). Moreover, using interrupted time-series analyses, the study in France estimated that most of the reduction in IPD was due to reductions in influenza and RSV, with no association with pneumococcal carriage rates.

Importantly, we observed a substantial decline in overall pneumococcal carriage density after the implementation of NPIs, which was driven by reductions in capsular pneumococcal carriage density. There was no corresponding reduction in nonencapsulated pneumococcal carriage density. Capsular pneumococcal carriage density decreased by approximately 70 to 90% in the two periods with NPIs compared with the pre-COVID-19 period. In contrast, the only other study to evaluate the impact of NPIs on pneumococcal carriage density found no difference pre- and post-NPI implementation in Israel using a semiquantitative plate scoring method (4). These differences may be partially explained by the use of the more sensitive quantitative PCR method in our study, as well as differences in study settings such as NPI stringency. It is unclear why there was no reduction in nonencapsulated carriage density in our study. Interestingly, we have previously found that coinfection of infant mice with a murine analogue of RSV increased the carriage density of capsulated pneumococci, but this increase was not observed in nonencapsulated pneumococci (15). Given the reductions in circulating respiratory viruses detected in many settings, it is possible that nonencapsulated pneumococcal carriage density was less affected if respiratory viruses (that act to increase pneumococcal carriage density) largely act on capsulated pneumococci.

A major strength of this study is the use of carriage data from healthy children. Many studies investigating the impact of NPIs on non-SARS-CoV-2 pathogens rely on disease data, which can be influenced by behavioral changes such as reluctance to seek medical treatment for fear of contracting COVID-19. The use of sensitive, fully quantitative, serotyping methods (16) enabled us to quantify the impact of NPIs on pneumococci, and to evaluate differences in capsular and nonencapsulated pneumococci which has not been investigated previously. This study was not able to evaluate the impact of NPIs on pneumococcal carriage acquisition given the cross-sectional study design. Longitudinal carriage studies conducted during the COVID-19 pandemic could provide important data on the impact of NPIs on pneumococcal carriage acquisition and any associated impact on pneumococcal carriage density. In the absence of pneumococcal carriage acquisition data, assessment of multiple serotype carriage may provide insight into changes in pneumococcal acquisition. We found multiple serotype carriage was infrequent and similar across all periods, indicating little evidence of a change in acquisition in this study. Additionally, other temporal changes may have occurred during the study that were not accounted for.

The implementation of NPIs has been associated with declines in IPD (3), which has been temporally associated with the suppression of RSV, influenza, and human metapneumovirus (4, 6). In this study, there was a reduction in symptoms of upper respiratory tract infection and a decline in the use of antibiotics during the NPI periods compared with the pre-COVID period, which is consistent with a reduction in respiratory infections. Respiratory viruses are associated with increased pneumococcal carriage density (8, 9, 17), which is a risk factor for disease (9, 13, 18). The decline in pneumococcal carriage density associated with NPI implementation therefore provides a plausible explanation for the reductions in IPD that have been observed elsewhere in the absence of a substantive reduction in pneumococcal carriage prevalence. For example, NPI implementation may result in reductions in circulating respiratory viruses that act to increase pneumococcal carriage density. In the absence of these viruses, pneumococcal carriage density would be lower, leading to a reduced chance of developing IPD. Interventions that target respiratory viruses therefore have the potential to reduce IPD, increasing the cost-effectiveness and impact of viral prevention and treatment strategies. Further research examining the links between respiratory viruses and pneumococcal carriage acquisition, carriage prevalence, and carriage density is needed to fully elucidate the impact NPIs have on IPD.

## MATERIALS AND METHODS

### Study design and participants.

Nasopharyngeal samples were collected from children aged 24 months between 25 December 2018 and 18 June 2020 as part of a trial investigating reduced-dose schedules of pneumococcal conjugate vaccines (PCV) in Ho Chi Minh City, Vietnam (19). In the trial, healthy children from the community who were born at ≥36 weeks gestation and had no significant maternal or perinatal history were enrolled at 2 months of age and followed up to 24 months of age. Further details on inclusion and exclusion criteria are provided in the trial protocol (19). Participants were randomized to one of five groups and received PCV10 at 12 months (0 + 1) or at 2 and 12 months (1 + 1), PCV13 at 12 months (0 + 1) or at 2 and 12 months (1 + 1), or a single dose of PCV10 at 24 months after the 24-month sample collection (unvaccinated comparator). Nasopharyngeal sample collection for the 24-month time point occurred before and after the emergence of SARS-CoV-2, enabling the impact of NPIs on pneumococcal carriage prevalence and density to be evaluated. All children with a 24-month sample were eligible for inclusion in this secondary analysis.

### Ethics statements.

Ethical approval was obtained for the vaccine trial from the Human Research Ethics Committee of the Royal Children’s Hospital Melbourne, the Institutional Review Board at the Pasteur Institute of Ho Chi Minh City, and the Vietnam Ministry of Health Ethical Review Committee for Biomedical Research. The vaccine trial is registered at clinicaltrials.gov (NCT03098628). Written informed consent was obtained from participants.

All participants gave consent for results to be published in scientific journals as part of the Participant Information and Consent form completed during enrollment into the vaccine trial.

### Laboratory analyses and carriage outcomes.

Swabs were collected and stored according to WHO guidelines (20). Pneumococcal carriage prevalence and density were determined by quantitative PCR (qPCR) targeting the *lytA* gene and molecular serotyping using Senti-SPv1.5 DNA microarrays (BUGS Bioscience), with analysis using a custom web-based software (19, 21).

Serotype 11F-like was reported as 11A, and serotypes 15B and 15C were reported as 15B/C (22, 23). Overall pneumococcal carriage prevalence was defined as carriage of any pneumococci. Capsular carriage included carriage of any serotype excluding nonencapsulated pneumococci. Nonencapsulated carriage included carriage of previously described nonencapsulated pneumococci, including NT2, NT3b, NT4a, and NT4b (24). Any sample containing pneumococci was considered positive. For example, a sample containing both capsular and nonencapsulated serotypes was considered positive for overall, capsular, and nonencapsulated carriage prevalence.

### NPI periods.

Three time periods were defined based on key events and the implementation of NPIs in Vietnam (25). The pre-COVID-19 period was defined from the start of the 24-month sample collection until the World Health Organization was notified of a cluster of cases in Wuhan, China (25 December 2018 to 31 December 2019). The first NPI period included school closures, warning messages to the public, restrictions on nonessential businesses, and a mask wearing mandate (NPI-1, 03 February 2020 to 31 March 2020). A 3-week lockdown was implemented in Ho Chi Minh City from 01 April to 22 April 2020, during which time study clinics were closed and no samples were collected. The second NPI period started after the lockdown and concluded at the end of sample collection (NPI-2, 23 April 2020 to 18 June 2020). During NPI-2, the mask wearing mandate continued and there was a staggered reopening of nonessential businesses (25, 26). Samples collected between 1 January 2020 and 2 February 2020 were excluded from analyses, as preventative measures (such as mask wearing, hand hygiene, and self-isolation) may have been implemented before such policies were officially introduced.

### Statistical analyses.

We determined the prevalence and density of overall, capsular, and nonencapsulated pneumococcal carriage, and plotted serotype-specific pneumococcal carriage prevalence and density. Participant characteristics were compared between time periods using the chi-squared test for comparisons of proportions, or quantile regression with bootstrapped standard errors for comparisons of medians. Potential confounders were assessed using a directed acyclic graph, with no confounders identified (see Fig. S1 in the supplemental material). Pneumococcal carriage density data were log_10_-transformed and are expressed as log_10_ genome equivalents per mL (log_10_ GE/mL). Serotype-specific carriage density was calculated by multiplying the overall pneumococcal load (as determined by qPCR) by the corresponding relative abundance of the serotype (as determined by DNA microarray).

In the primary analyses, pneumococcal carriage prevalence and density in each NPI period were compared with the pre-COVID-19 period using unadjusted log-binomial and linear regression. Log-binomial regression was used to compare carriage prevalence across time periods, with results reported as prevalence ratios, 95% confidence intervals (CIs) and *P* values. Linear regression was used to compare log_10_-transformed pneumococcal carriage density across time periods in pneumococcal carriers, with results reported as differences in means, 95% CIs, and *P* values. Percentage changes in pneumococcal carriage density were calculated by (10^β^^^^ − 1) × 100%, where β^^^ is the estimate of the linear regression coefficient, representing the difference in means of log_10_-transformed pneumococcal carriage density in the NPI period compared with the pre-COVID-19 period. Regression analyses were conducted for overall, capsular, and nonencapsulated pneumococcal carriage prevalence and density outcomes.

Three additional analyses were conducted: (i) to evaluate the direct effect of the NPI period on pneumococcal carriage prevalence and density (i.e., the effect not via intermediate variables), an analysis was conducted including district and season in the models; (ii) to evaluate the potential impact of including children with symptoms of upper respiratory tract infection (URTI) in the primary analysis, an analysis was conducted restricting to children without any self-reported symptoms of URTI; (iii) to evaluate whether any associations observed in the primary analysis were independent of the PCV trial intervention, an analysis was conducted restricting to unvaccinated children alone. Statistical analyses were conducted using Stata v16.0 (StataCorp LLC).

### Data availability.

Data will be stored in the Bill and Melinda Gates Foundation Knowledge Integration (KI) repository.

## References

[1] Weiser JN, Ferreira DM, Paton JC. 2018. *Streptococcus pneumoniae*: transmission, colonization and invasion. Nat Rev Microbiol 16:355–367. doi:10.1038/s41579-018-0001-8. 29599457 PMC5949087

[2] Baker RE, Park SW, Yang W, Vecchi GA, Metcalf CJE, Grenfell BT. 2020. The impact of COVID-19 nonpharmaceutical interventions on the future dynamics of endemic infections. Proc Natl Acad Sci USA 117:30547–30553. doi:10.1073/pnas.2013182117. 33168723 PMC7720203

[3] Brueggemann AB, Jansen van Rensburg MJ, Shaw D, McCarthy ND, Jolley KA, Maiden MCJ, van der Linden MPG, Amin-Chowdhury Z, Bennett DE, Borrow R, Brandileone M-CC, Broughton K, Campbell R, Cao B, Casanova C, Choi EH, Chu YW, Clark SA, Claus H, Coelho J, Corcoran M, Cottrell S, Cunney RJ, Dalby T, Davies H, de Gouveia L, Deghmane A-E, Demczuk W, Desmet S, Drew RJ, Du Plessis M, Erlendsdottir H, Fry NK, Fuursted K, Gray SJ, Henriques-Normark B, Hale T, Hilty M, Hoffmann S, Humphreys H, Ip M, Jacobsson S, Johnston J, Kozakova J, Kristinsson KG, Krizova P, Kuch A, Ladhani SN, Lâm T-T, Lebedova V, et al. 2021. Changes in the incidence of invasive disease due to *Streptococcus pneumoniae*, *Haemophilus influenzae*, and *Neisseria meningitidis* during the COVID-19 pandemic in 26 countries and territories in the Invasive Respiratory Infection Surveillance Initiative: a prospective analysis of surveillance data. Lancet Digit Health 3:e360–e370. doi:10.1016/S2589-7500(21)00077-7. 34045002 PMC8166576

[4] Danino D, Ben-Shimol S, van der Beek BA, Givon-Lavi N, Avni YS, Greenberg D, Weinberger DM, Dagan R. 2021. Decline in pneumococcal disease in young children during the Coronavirus disease 2019 (COVID-19) pandemic in Israel associated with suppression of seasonal respiratory viruses, despite persistent pneumococcal carriage: a prospective cohort study. Clinical Infectious Diseases Dec 75:e1154–e1164. doi:10.1093/cid/ciab1014. PMC8754767 34904635

[5] Willen L, Ekinci E, Cuypers L, Theeten H, Desmet S. 2021. Infant pneumococcal carriage in Belgium not affected by COVID-19 containment measures. Front Cell Infect Microbiol 11:825427. doi:10.3389/fcimb.2021.825427. 35111700 PMC8801737

[6] Rybak A, Levy C, Angoulvant F, Auvrignon A, Gembara P, Danis K, Vaux S, Levy-Bruhl D, van der Werf S, Béchet S, Bonacorsi S, Assad Z, Lazzati A, Michel M, Kaguelidou F, Faye A, Cohen R, Varon E, Ouldali N. 2022. Association of nonpharmaceutical interventions during the COVID-19 pandemic with invasive pneumococcal disease, pneumococcal carriage, and respiratory viral infections among children in France. JAMA Netw Open 5:e2218959. doi:10.1001/jamanetworkopen.2022.18959. 35763298 PMC9240903

[7] Petrović V, Milosavljević B, Djilas M, Marković M, Vuković V, Andrijević I, Ristić M. 2022. Pneumococcal nasopharyngeal carriage in children under 5 years of age at an outpatient healthcare facility in Novi Sad, Serbia during the COVID-19 pandemic. IJID Reg 4:88–96. doi:10.1016/j.ijregi.2022.07.001. 35865274 PMC9294645

[8] McCullers JA, McAuley JL, Browall S, Iverson AR, Boyd KL, Henriques Normark B. 2010. Influenza enhances susceptibility to natural acquisition of and disease due to *Streptococcus pneumoniae* in ferrets. J Infect Dis 202:1287–1295. doi:10.1086/656333. 20822454 PMC3249639

[9] Wolter N, Tempia S, Cohen C, Madhi SA, Venter M, Moyes J, Walaza S, Malope-Kgokong B, Groome M, Du Plessis M, Magomani V, Pretorius M, Hellferscee O, Dawood H, Kahn K, Variava E, Klugman KP, von Gottberg A. 2014. High nasopharyngeal pneumococcal density, increased by viral coinfection, is associated with invasive pneumococcal pneumonia. J Infect Dis 210:1649–1657. doi:10.1093/infdis/jiu326. 24907383

[10] Diavatopoulos DA, Short KR, Price JT, Wilksch JJ, Brown LE, Briles DE, Strugnell RA, Wijburg OL. 2010. Influenza A virus facilitates *Streptococcus pneumoniae* transmission and disease. FASEB J 24:1789–1798. doi:10.1096/fj.09-146779. 20097876

[11] Chappell KJ, Brealey JC, Mackay IM, Bletchly C, Hugenholtz P, Sloots TP, Sly PD, Young PR. 2013. Respiratory syncytial virus infection is associated with increased bacterial load in the upper respiratory tract in young children. J Med Microbiol Diagn 01 S:1–9.

[12] Short KR, Reading PC, Wang N, Diavatopoulos DA, Wijburg OL. 2012. Increased nasopharyngeal bacterial titers and local inflammation facilitate transmission of *Streptococcus pneumoniae*. mBio 3:e00255-12. doi:10.1128/mBio.00255-12. 23015738 PMC3518912

[13] Alpkvist H, Athlin S, Nauclér P, Herrmann B, Abdeldaim G, Slotved H-C, Hedlund J, Strålin K. 2015. Clinical and microbiological factors associated with high nasopharyngeal pneumococcal density in patients with pneumococcal pneumonia. PLoS One 10:e0140112. doi:10.1371/journal.pone.0140112. 26466142 PMC4605601

[14] Keller LE, Robinson DA, McDaniel LS. 2016. Nonencapsulated *Streptococcus pneumoniae*: emergence and pathogenesis. mBio 7:e01792. doi:10.1128/mBio.01792-15. 27006456 PMC4807366

[15] Manna S, McAuley J, Jacobson J, Nguyen CD, Ullah MA, Sebina I, Williamson V, Mulholland EK, Wijburg O, Phipps S, Satzke C. 2022. Synergism and antagonism of bacterial-viral coinfection in the upper respiratory tract. mSphere 7:e0098421. doi:10.1128/msphere.00984-21. 35044807 PMC8769199

[16] Satzke C, Dunne EM, Porter BD, Klugman KP, Mulholland EK, Group P project. 2015. The PneuCarriage Project: a multi-centre comparative study to identify the best serotyping methods for examining pneumococcal carriage in vaccine evaluation studies. PLoS Med 12:e1001903. doi:10.1371/journal.pmed.1001903. 26575033 PMC4648509

[17] Parker AM, Jackson N, Awasthi S, Kim H, Alwan T, Wyllie AL, Baldwin AB, Brennick NB, Moehle EA, Giannikopoulos P, Kogut K, Holland N, Mora-Wyrobek A, Eskenazi B, Riley LW, Lewnard JA. 2022. Association of upper respiratory *Streptococcus pneumoniae* colonization with SARS-CoV-2 infection among adults. Clin Infect Dis:ciac907. doi:10.1093/cid/ciac907. 36401872

[18] Carr OJJ, Vilivong K, Bounvilay L, Dunne EM, Lai JYR, Chan J, Vongsakid M, Changthongthip A, Siladeth C, Ortika B, Nguyen C, Mayxay M, Newton PN, Mulholland K, Do LAH, Dubot-Pérès A, Satzke C, Dance DAB, Russell FM. 2022. Nasopharyngeal pneumococcal colonization density is associated with severe pneumonia in young children in the Lao PDR. J Infect Dis 225:1266–1273. doi:10.1093/infdis/jiab239. 33974708 PMC8974848

[19] Temple B, Tran HP, Dai VTT, Bright K, Uyen DY, Balloch A, Licciardi P, Nguyen CD, Satzke C, Smith-Vaughan H, Nguyen TV, Mulholland K. 2021. Simplified 0+1 and 1+1 pneumococcal vaccine schedules in Ho Chi Minh City, Vietnam: protocol for a randomised controlled trial. BMJ Open 11:e056505. doi:10.1136/bmjopen-2021-056505. PMC8634020 34845082

[20] Satzke C, Turner P, Virolainen-Julkunen A, Adrian PV, Antonio M, Hare KM, Henao-Restrepo AM, Leach AJ, Klugman KP, Porter BD, Sá-Leão R, Scott JA, Nohynek H, O'Brien KL, WHO Pneumococcal Carriage Working Group. 2013. Standard method for detecting upper respiratory carriage of *Streptococcus pneumoniae*: updated recommendations from the World Health Organization Pneumococcal Carriage Working Group. Vaccine 32:165–179. doi:10.1016/j.vaccine.2013.08.062. 24331112

[21] Newton R, Hinds J, Wernisch L. 2011. Empirical Bayesian models for analysing molecular serotyping microarrays. BMC Bioinformatics 12:88. doi:10.1186/1471-2105-12-88. 21453458 PMC3076268

[22] van Selm S, van Cann LM, Kolkman MAB, van der Zeijst BAM, van Putten JPM. 2003. Genetic basis for the structural difference between *Streptococcus pneumoniae* serotype 15B and 15C capsular polysaccharides. Infect Immun 71:6192–6198. doi:10.1128/IAI.71.11.6192-6198.2003. 14573636 PMC219561

[23] Manna S, Ortika BD, Dunne EM, Holt KE, Kama M, Russell FM, Hinds J, Satzke C. 2018. A novel genetic variant of *Streptococcus pneumoniae* serotype 11A discovered in Fiji. Clin Microbiol Infect 24:428.e1–428.e7. doi:10.1016/j.cmi.2017.06.031. PMC5869949 28736074

[24] Salter SJ, Hinds J, Gould KA, Lambertsen L, Hanage WP, Antonio M, Turner P, Hermans PWM, Bootsma HJ, O'Brien KL, Bentley SD. 2012. Variation at the capsule locus, cps, of mistyped and non-typable *Streptococcus pneumoniae* isolates. Microbiology (Reading) 158:1560–1569. doi:10.1099/mic.0.056580-0. 22403189 PMC3541774

[25] Nguyen TV, Tran QD, Phan LT, Vu LN, Truong DTT, Truong HC, Le TN, Vien LDK, Nguyen TV, Luong QC, Pham QD. 2021. In the interest of public safety: rapid response to the COVID-19 epidemic in Vietnam. BMJ Glob Health 6:e004100. doi:10.1136/bmjgh-2020-004100. PMC7839307 33495284

[26] Pham QD, Stuart RM, Nguyen Tv, Luong QC, Tran QD, Pham TQ, Phan LT, Dang TQ, Tran DN, Do HT, Mistry D, Klein DJ, Abeysuriya RG, Oron AP, Kerr CC. 2021. Estimating and mitigating the risk of COVID-19 epidemic rebound associated with reopening of international borders in Vietnam: a modelling study. Lancet Glob Health 9:e916–e924. doi:10.1016/S2214-109X(21)00103-0. 33857499 PMC8041387

[27] Beck HE, Zimmermann NE, McVicar TR, Vergopolan N, Berg A, Wood EF. 2018. Present and future köppen-geiger climate classification maps at 1-km resolution. Sci Data 5:180214. doi:10.1038/sdata.2018.214. 30375988 PMC6207062

