# Supplementary material for: Impact of COVID-19 Nonpharmaceutical Interventions on Pneumococcal Carriage Prevalence and Density in Vietnam
Source: Microbiol Spectr. 2023 Jan 16;11(1):e03615-22. doi: 10.1128/spectrum.03615-22 (PMC9927266; doi:10.1128/spectrum.03615-22)
Supplement: Supplemental file 1 — Fig. S1 to S3 and Tables S1 to S3. Download spectrum.03615-22-s0001.pdf, PDF file, 0.3 MB [file spectrum.03615-22-s0001.pdf]

Impact of COVID-19 non-pharmaceutical interventions on pneumococcal carriage prevalence and density in Vietnam

SUPPLEMENTARY INFORMATION

Directed acyclic graph (DAG)

A DAG was constructed to evaluate the relationships between the exposure (NPI period), outcomes (pneumococcal carriage prevalence or pneumococcal carriage density), and other key factors. Arrows are used to indicate the direction of the association. Green lines indicate causal pathways, and red lines indicate biasing pathways (none identified). Measured variables are shown in dark grey and unmeasured variables are shown in light grey. No potential confounders were identified for the primary analysis, and therefore no covariates were included for adjustment. As the DAG indicates there are some intermediate variables on the causal pathway between exposure and outcome (i.e. some of the effect of NPI period on pneumococci could be mediated by district of residence and season), we chose to conduct an additional analysis evaluating the direct effect of NPI period on pneumococcal carriage and density. In the additional analysis, district of residence and season were included in the models for adjustment.

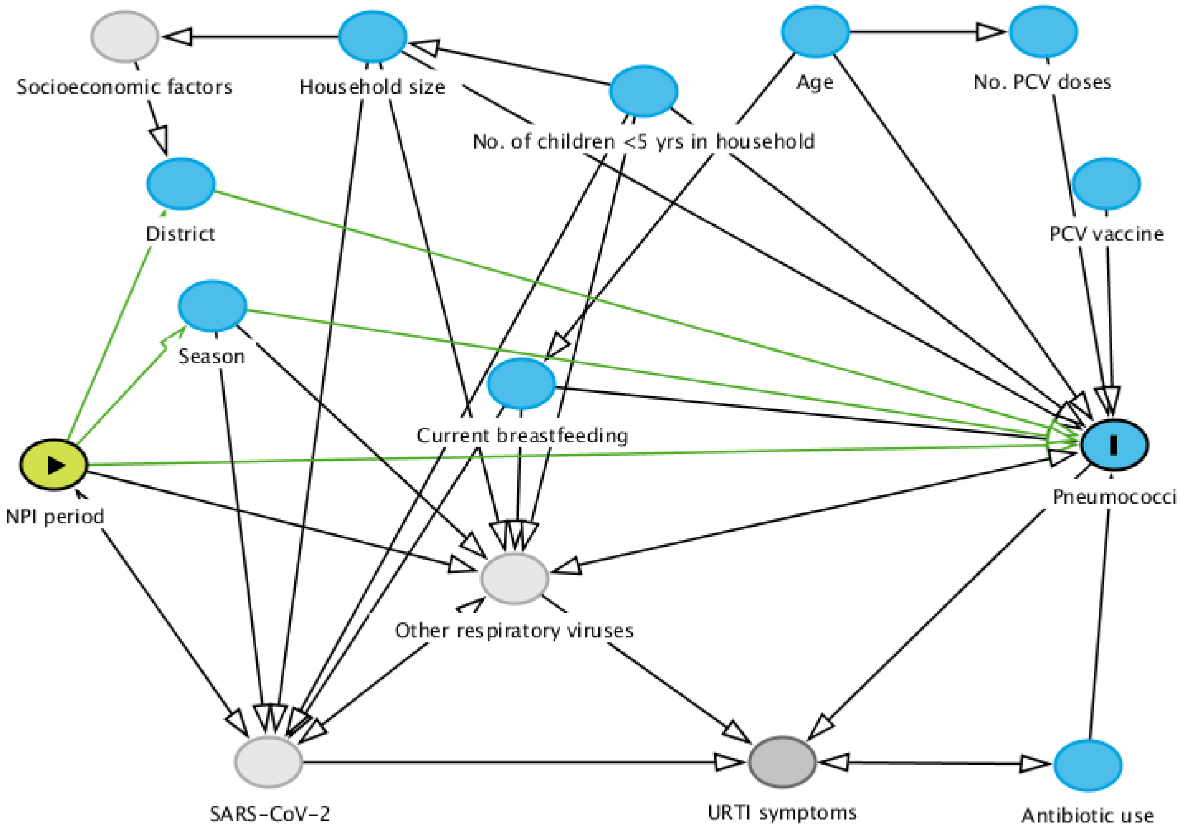

Figure S1. Directed acyclic graph depicting the relationships between the exposure (NPI period), outcomes (pneumococcal carriage or density), and other key factors. Green lines indicate causal pathways, and a red line would indicate a biasing pathway (none identified). Measured variables are shown in dark grey and unmeasured variables are shown in light grey.

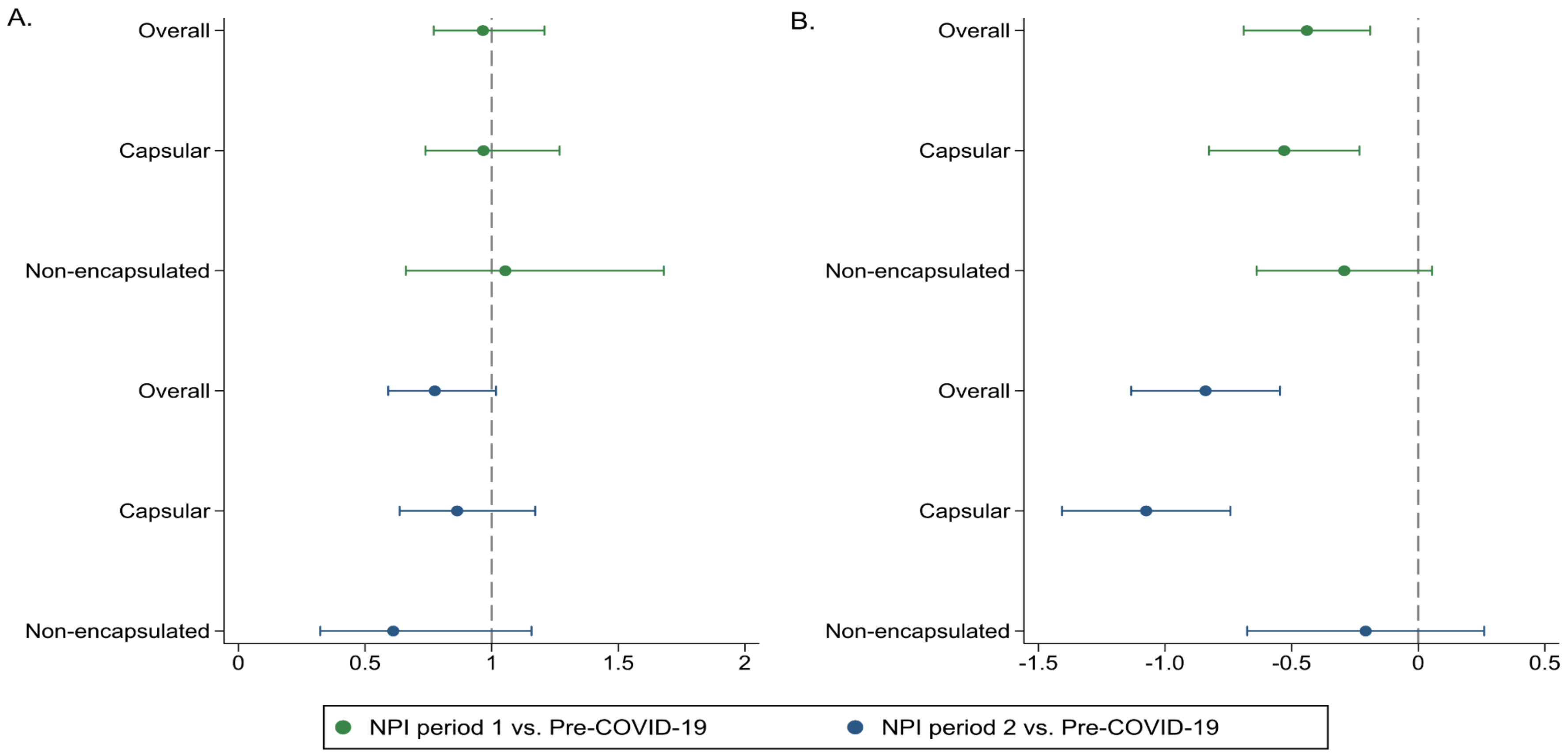

Figure S2. Pneumococcal carriage A) prevalence ratios (95% CIs), and B) difference in density means (95% CIs) in periods with non-pharmaceutical interventions (NPIs) compared with the pre-COVID-19 period.

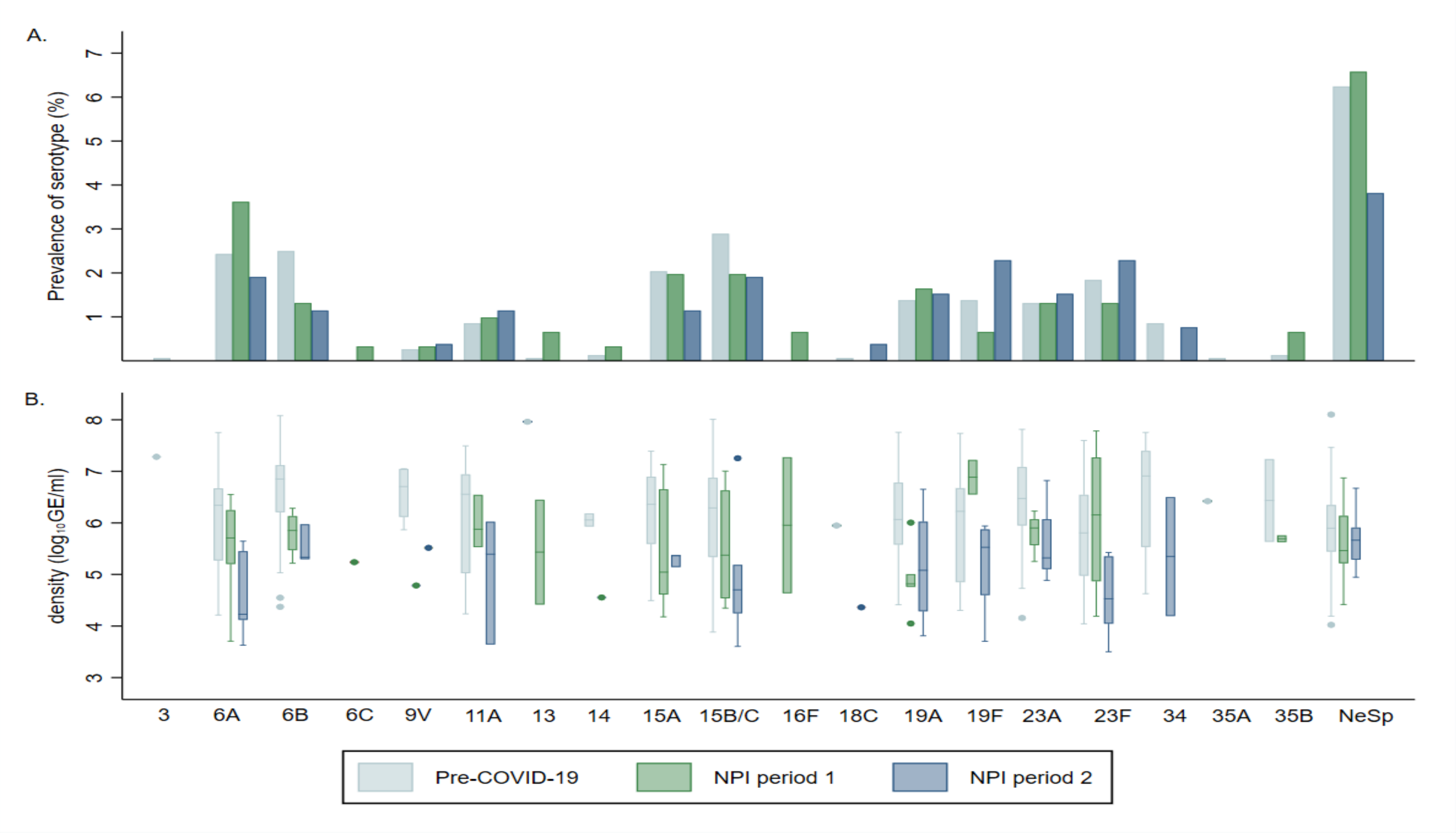

Figure S3. Serotype-specific A) pneumococcal carriage prevalence, and B) pneumococcal carriage density (log_{10} genome equivalents per mL) among pneumococcal carriers in periods with non-pharmaceutical interventions (NPIs) compared with the pre-COVID-19 period. Boxes in B) depict interquartile range (IQR) with a central line for the median. Data points further than the 25th/75th percentile plus 1.5 times the IQR are plotted as individual points. COVID-19= coronavirus disease 2019. NeSp= non-encapsulated *Streptococcus pneumoniae.*

Table S1. Adjusted coefficients and 95% confidence intervals for pneumococcal carriage prevalence and density in periods with non-pharmaceutical interventions (NPIs) compared with the pre-COVID-19 period. Coefficients are prevalence ratios for pneumococcal carriage and difference in means for density.

Outcome*	N	NPI period 1 Coefficient (95% CI)	p-value	NPI period 2 Coefficient (95% CI)	p-value
Carriage					
Overall	2106	0.84 (0.65 to 1.08)	0.171	0.81 (0.61 to 1.07)	0.140
Capsular§	2088	0.82 (0.61 to 1.11)	0.201	0.90 (0.66 to 1.24)	0.525
Non-encapsulated§	2088	0.99 (0.58 to 1.70)	0.967	0.64 (0.33 to 1.23)	0.178
Density†					
Overall	481	-0.35 (-0.64 to -0.06)	0.017	-0.85 (-1.16 to -0.55)	<0.001
Capsular§	361	-0.39 (-0.73 to -0.05)	0.025	-1.11 (-1.46 to -0.76)	<0.001
Non-encapsulated§	125	-0.31 (-0.72 to 0.10)	0.133	-0.21 (-0.69 to 0.28)	0.394

*Adjusted for district of residence and season of swab collection to evaluate the direct effect of the NPI period on pneumococci.
§Serotype-specific data were not available for n=18 samples (n=15 pre-COVID-19, n=3 NPI-1).
†Assessed in pneumococcal carriers only and reported in $\log_{10}$ genome equivalents per mL.
95% CI= 95% confidence interval.

Table S2. Unadjusted coefficients and 95% confidence intervals for pneumococcal carriage prevalence and density in participants without symptoms of upper respiratory tract infection in periods with non-pharmaceutical interventions (NPIs) compared with the pre-COVID-19 period. Coefficients are prevalence ratios for pneumococcal carriage and difference in means for density.

Outcome	N	NPI period 1 Coefficient (95% CI)	p-value	NPI period 2 Coefficient (95% CI)	p-value
Carriage – without symptoms of URTI					
Overall	1835	1.04 (0.82 to 1.31)	0.758	0.81 (0.61 to 1.07)	0.132
Capsular§	1821	1.04 (0.78 to 1.37)	0.796	0.92 (0.67 to 1.26)	0.605
Non-encapsulated§	1821	1.06 (0.65 to 1.72)	0.819	0.57 (0.29 to 1.12)	0.103
Density† – without symptoms of URTI					
Overall	402	-0.40 (-0.66 to -0.14)	0.002	-0.81 (-1.11 to -0.51)	<0.001
Capsular§	301	-0.47 (-0.78 to -0.16)	0.003	-1.00 (-1.34 to -0.66)	<0.001
Non-encapsulated§	107	-0.24 (-0.61 to 0.12)	0.193	-0.24 (-0.75 to 0.26)	0.347

§Serotype-specific data were not available for n=14 samples (n=11 pre-COVID-19, n=3 NPI-1).
†Assessed in pneumococcal carriers only and reported in log_{10} genome equivalents per mL.
95% CI= 95% confidence interval. URTI= upper respiratory tract infection.

Table S3. Unadjusted coefficients and 95% confidence intervals for pneumococcal carriage prevalence and density in unvaccinated participants in periods with non-pharmaceutical interventions (NPIs) compared with the pre-COVID-19 period. Coefficients are prevalence ratios for pneumococcal carriage and difference in means for density.

Outcome	N	NPI period 1 Coefficient (95% CI)	p-value	NPI period 2 Coefficient (95% CI)	p-value
Carriage – unvaccinated only					
Overall	737	1.23 (0.89 to 1.69)	0.212	1.08 (0.74 to 1.58)	0.687
Capsular§	730	1.24 (0.84 to 1.82)	0.278	1.29 (0.86 to 1.95)	0.224
Non-encapsulated§	730	1.12 (0.51 to 2.47)	0.782	0.40 (0.10 to 1.63)	0.199
Density† – unvaccinated only					
Overall	188	-0.39 (-0.78 to 0.00)	0.048	-0.82 (-1.27 to -0.37)	<0.001
Capsular§	146	-0.39 (-0.86 to 0.07)	0.099	-0.98 (-1.48 to -0.48)	<0.001
Non-encapsulated§	40	-0.15 (-0.66 to 0.36)	0.557	0.02 (-0.87 to 0.91)	0.966

§Serotype-specific data were not available for n=7 samples (n=5 pre-COVID-19, n=2 NPI-1).
†Assessed in pneumococcal carriers only and reported in log_{10} genome equivalents per mL.
95% CI= 95% confidence interval.
